# Third Annual Meeting of the American Dental Convention

**Published:** 1857-12

**Authors:** 


### THIRD ANNUAL MEETING OF THE AMERICAN DENTAL CONVENTION.

It is probable that by this time the reading members of the profession have perused the report of the discussions at the Boston meeting. It is not our purpose to review, or to notice at length, all the various discussions contained in the report, but only such points as need farther elucidation, or are calculated to mislead. And while we proceed, we will still bear in mind that it is impossible, in a condensed report, faithfully to represent the speakers. Hence we will hold no one strictly accountable for any remarks that may be imputed to him.

It is a matter much to be regretted that the "Business Committee" failed—worse than failed to perform the principal duty assigned them. That committee was definitely instructed to report "the order of business," at least, as far as subjects for discussion were concerned, through the periodicals, at an early day. For this failure, they may be justly held accountable for much of the hap-hazard discussion contained in the report. It is not to be expected that men can speak as directly to the point, and as profitably to themselves and others, when totally unprepared, as when they have carefully studied the subjects before them, and selected the language in which to present their views of them. Their report *at the meeting* we regard as altogether out of time, and would suggest to their successors, that, if they can not report till the convention meets, they refrain altogether; for if the convention must be taken by surprise, it can select its subjects better than any committee can *hastily* do it for them.

As usual in mass meetings, considerable time appears to have been taken up at the start with unimportant matters, or important matters, if you please, *unimportantly* handled. For instance, Dr. Weiber sends a specimen of sponge gold, —the president suggests its reference to a committee. Dr. F. moves that that committee be the officers of the convention, Dr. T. argues at length against referring it to a committee, but seems to be in favor of referring it to the officers. He tells us, " the mere fact of the appointment of a committee would be enough in some cases, to afford an opportunity for parties to go home and claim credit for themselves, on the ground that the convention had considered their inventions of sufficient importance to appoint a committee to investigate them. He wished to avoid all such difficulties."

It is hard to see why the reference of the subject to a committee of officers is less respectful than its reference to a committee of privates; but we suppose such is the case, and it was modest in Dr. T., an officer, to make the discovery.

"Dr. D. said they must be careful to avoid either extreme." That is, according to the report, its reference to the officers is one extreme, to a committee of private members is the other. Dr. F., Dr. S., Dr. B., and Dr. A. continue the discussion, the latter being afraid, like Dr. T., of establishing a precedent, or of doing something that would induce somebody to do something else. We only wish that all such discussion could be suppressed from the report. It is the height of folly to be afraid to act lest others take undue advantage of our action; for if we remain idle, they will take like advantage of our idleness. And as to " precedent," it is the greatest humbug of the age, unless it be its kindred idea, " consistency." If an action be wrong in itself, the fact of our having performed it yesterday is no argument, nor even an excuse for its performance to-day. And if an action be right and expedient, to refrain from doing it because Tom or Bob may be benefited by it, even by dishonorable appropriation, is like the conduct of the man who sits in darkness, lest others might enjoy his lamplight; and the feeling which prompts such argument is like that of the man who cannot enjoy a sleigh-ride, because the little boy hitches on his hand-sled and steals (?) a happy ride at his expense.

The question of membership again came up, on inquiry, from a gentleman in the hall. It is strange that this subject was not understood after the thorough sifting it received at New York; yet we find that not all those present at that discussion had clear views of the conclusions arrived at. The remarks of Professor Taylor, and the action which followed, have, no doubt put a *quietus* on this subject.

The constitution being totally thrown aside, the Convention became simply a "mass meeting," and, as such, it adjourned to meet at Cincinnati. As there is no provision for a change of officers, the constitution being gone, we presume the present incumbents will have the opportunity to enjoy official honors to their heart's content. It is probable that something "like a piece of red tape to tie up the members" will be found rather convenient, after all.

In the discussion of the first subject presented by the business committee, the range of debate takes a singular turn. The proverb says, "Straws show which way the wind blows;" but in this case, a straw showed the wind which way to blow. We allude to the paper "on the effects of saleratus, cream of tarter, and carbonate of soda,"—certainly as light as a straw when compared with what might and *could* have been said on the subject. The reading of the paper was, perhaps, well enough; but, unfortunately, almost the entire discussion of this subject runs in the same channel. The author of the paper regards the use of alkalies in making bread, as the principal cause of diseased teeth: and the report represents himself and others as classifying the three substances named in the paper with the alkalies. The question is asked, "Is saleratus a pure alkali?" Each of the three substances named is a salt. Two of them, it is true, are alkaline carbonates; and as carbonic acid, on account of its elasticity, is easily displaced, they often act the part of alkalies. The other is a *bi-tartrate*, and has therefore an acid re-action. When brought in contact with either of the others, the bitartrate is converted into a *tartrate*, and the carbonic acid, of course, escapes as gas. If carbonate of soda be used with it, Rochelle salt, the tartrate, potash and soda, is formed, which is a mild, cooling laxative and diuretic." If saleratus be used, the salt formed is the neutral tartrate of potash, as harmless and pleasant in its effects as the other. As far, then, as acids and alkalies are concerned, they are neutralized before the bread is eaten; hence to agonize over *their* action on the teeth is illegitimate sympathy. And if he looks after the neutral salts formed by the reactions, he will find the quantity so small that it will neither reward his research nor annoy his patient. Why does not the author attack another bread formula? A very beautiful and healthy bread is prepared by mixing his hated bi-carbonate of soda and *hydro-chloric* acid—that acid which is pre-eminently the destroying angel of the dental organs. Now this may look frightful to the author; but it is only seasoning the bread with common salt.

But, says the author, if the acids and alkalies do neutralize each other, they destroy "the goodness of the wheat, and *might, in this way, act upon the teeth.*" Goodness! (?) If a rattlesnake had been in the knot-hole where the bull-frog was when the loafer stood by it fishing, *it might have bit him.*

With the remarks of Professor Harris on this part of the discussion we regret that we can not agree, or rather, we regret that Professor H. is made to utter sentiments which we regard as evidently unsound. He says, "Again, they oftentimes meet with instances of caries, or erosion, if they pleased to call it such, immediately *after* the eruption of the teeth from the gums, showing that it was to the action of the fluid contained in the sacs of the teeth, and that, too, after they had become completely enamelled, that decomposition was attributable."

To our mind it is not very clear that erosion discovered *after* the teeth emerge through the gums shows that it existed *before* their eruption. It is well known that the teeth are exposed to the influence of external agents before they have progressed sufficiently to be examined by ocular inspection. And it should be well known that the acids which cause "erosion" act with such energy that but little time is required to produce observable effects. Did Professor H. ever find this erosion *before* "the eruption of the teeth from the gums?" Till that is done his conclusion is based on a mere inference, and one that is at least doubtfully deducible from the premises. As far as we are aware, nothing similar is found in the entire system. Why do the synovial fluids not act on the extremities of the bones in the same persons, or in those of similar constitution? Erosion would be more readily produced here than on the enamel. If the doctrine is true, we have a right to expect this; for Professor H. tells us that it depends "upon some peculiar constitutional idiosyncracy." And again he tells us that it is dependent "upon some peculiar cachetic habit of body." Now, if it arises from a *constitutional* idiosyncracy, how is it that this "cachetic habit of body" becomes so thoroughly *localized* that all the acid of the entire system is concentrated in the dental sacs? We are told, "he could call up a hundred examples of this kind;" but we are not referred to a single one discovered by post-mortem examination, where the teeth are not through the gum. The molars are particularly referred to as liable to erosion under these circumstances. Now, it is known to all, that, as these teeth are erupted, they are mainly covered by portions of the gum for some time after the fluids of the mouth have access to them. And it is obvious that the loose flaps of gum referred to, are just in the position which would retain these fluids in contact with the tooth till they become vitiated.

If a tooth be immersed in an acid solution capable of corroding it, all parts of its surface will be acted on. If the enamel be perfect in the depressions, it will not be corroded there more than on the cusps. But the erosion spoken of by Professor H. is seldom, if ever, found on the cusps. Now, if this erosion is due to the acidulation of the fluid contained in the sac, we have a tooth surrounded by, or immersed in, an acid solution, and yet it usually acts only on the depressions of the enamel. Why does it not attack the dentine and leave merely a shell of enamel to emerge through the gum ? This is the way acids do after the eruption of the tooth.

We are told that a somewhat analogous process occurs "in the destruction of the roots of the temporary teeth." And also, that "experiments had shown that the extraneous substance which is brought in contact with them, before the destructive process commences, exhales an acidulated fluid, which has a stronger affinity for the lime of the teeth, than the phosphoric, with which it is combined." What is the extraneous substance? And what acid does it exhale ? Did the same experiments demonstrate ? And does Professor H. wish to convey the idea that in erosion, caries, or even in the removal of the roots of the temporary teeth, the acting acid displaces the phosphoric, and takes the lime ? It is a fact well known that the acids which act most energetically on the teeth do not and can not *decompose* phosphate of lime.

In regard to the green trunk locks at Cape May, it is well known that an acid, alkaline, or saline atmosphere favors the oxydation of copper; and, as these locks were probably of *brass*, their change of color is not marvelous. The green substance was a compound of oxyd and chloride of copper. We do not think it probable, however, that the destruction of the teeth of sea-faring men is caused by the liberation of hydrochloric acid from the waters of the ocean. There is no evidence that the chloride of sodium is decomposed by simple evaporation. It sustains a red heat without decomposition, and, at a still higher heat, is only volatilized. It is true that if it should undergo decomposition, its liberated chlorine would take hydrogen from the water, and form hydrochloric acid. But, if the caries found in sailors' teeth is the result of the action of hydrochloric acid, which may be known by its appearance, we think its presence may be explained on a more correct hypothesis.* And in this connection, the suggestion of Dr. Dillingham is well worthy of notice. Having no opportunity of observing the teeth of sea-faring men, we do not know the character of the decay usually found in them, and consequently give no opinion as to what acid causes it.

The acid and alkali theory of Dr. B. is rather original. An excess of alkali in bread excites the stomach "to throw off an extra quantity of acid." Suppose it does, the alkali is there to neutralize it, and the stomach only does the cleverest thing it could, according to this theory. We are also told that "by taking alkalies into the stomach, we excite the production of an acid in the system—the acid thus produced acting prejudicially on the teeth." Now, of what does the stomach *make* this enormous, abnormal proportion of acid, by which it annihilates the excess of alkali, and still has a good stock left? It should be always remembered that the vital organs never violate chemical laws, and that it is not the most likely way to acidulate any liquid to add alkaline substances to it.

The decay found on the outside of the teeth, on a line with the gum, as described by Dr. Clark, is, no doubt, frequently caused by some acid which is found in the fluid exhaled by that portion of the gum. Often, when all foreign substances are removed from between the margin of the gum and the tooth, a properly prepared test paper inserted here will, in a short time, exhibit a decidedly acid reaction. What the acid is, we can not now say, having never obtained it in proper quantities for inspection. It is not probable, however, that the acid is peculiar to this place.

The remarks of Dr. A. on the effects of different kinds of food are, in general, well timed, and highly worthy of consideration; but, in conclusion, he finds it necessary to follow the fashion, and wind up with a general and indefinite statement

---

* See September Number, pp. 17, 18.

about "acids and alkalies," as if such substances must be composed of elements wholly foreign to the constitution.

We have some interesting and rather startling statistics respecting the teeth of the aboriginal tribes of our sea coasts. Dr. S. visited the sea coast of Georgia, "and *spent a day* in exhuming the remains which had been there thousands of years, for all he knew, and collected a bushel of teeth, among which he found only one case of disease."

We do not know how many teeth are contained in a bushel. Allowing two hundred to the pint, would give us 12,800 teeth; and if only adults were examined, we have, as a single day's work, the disinterment of 400 skeletons, and the personal inspection of their teeth. We are inclined to regard the doctor as the chief of resurrectioners, and presume that his achievements in this line will suffer no eclipse, till Gabriel himself commences operations in the same department.

As one of "the best means of securing a healthy denture," it is all-important to pay due attention to the condition of the mother during the periods of gestation and nursing, as suggested by Dr. P. and others. We often prescribe for the unborn offspring, with reference to the formation of the teeth, and are frequently most highly gratified with the results. Dentists who lack opportunity, and those who feel a delicacy in prescribing for such cases, may do much good, by suggestions through the family physician, who, if he be a gentleman, will thank them for the interest mansfested for his patients.

In constitutions which are defective in bony material, the use of sub-phosphate of lime, by the mother, from the commencement of ossification in uterus, till the eruption of the front teeth, has often a most happy influence on both mother and child. It invigorates the constitution of the former to such extent that she escapes many of the collateral ills which so frequently accompany gestation and lactation. It supplies an important element of ossific matter to the latter, and the usual result is well developed bones and good teeth.

The removal of the bran from flour, as referred to by Dr.

T., is, doubtless, a cause of defective osseous systems ; and it is true, as suggested by Dr. W., that the germ of the wheat contains more phosphoric acid than the hull. It is probable that the use of the entire grain would be more in accordance with chemical science, as applied to the laws of life. We admire the disposition to reach into matters, as manifested by Dr. W.

Of the discussion on "the best mode of filling teeth," we will say nothing at present, except merely to allude to the "dissolving quartz" filling, the thunder of which we are to hear, if not previously stolen, as soon as the material can be prevented from contracting as it crystallizes. Not being "disposed to steal one's thunder," nor to drive the lightning out of it, we will only say that "dissolving quartz" is nothing new, but that if it be forced to crystallize in solid masses of the desired size, then there will be a "new thing under the sun." If it can only be induced to expand a little in crystallizing, as Prof. Townsend tells us amalgams do, then we will certainly possess our heart's desire.

On the treatment of alveolar abscess, we have a strange confusion of terms, demonstrating the importance of Dr. Spaulding's suggestion in regard to "Dental Nomenclature." For instance, there is a very frequent use of the term *ulceration*, when *suppuration* is, undoubtedly, meant. A little attention would enable men to define their ideas with more clearness.

The injection of medicated agents into the sac, as suggested by Prof. Harris, is a very efficient mode of treatment. If a volatile escharotic be used, as creosote, its vapor may be readily passed into the sac and through the sinus, by closing the cavity of decay, after its insertion. This plan, first recommended by Dr. Ballard, we believe, is more convenient, and probably preferable to the other. When the discharge of pus is copious, it is not always proper to suppress it at once. By the method referred to, a gradually increased action may be brought to bear on it, by using different agents ; as, for example, alcohol, tincture of camphor, chloroform, and creosote, in the order named. With such treatment, if the constitution be in tolerable condition, we do not think "it would take, perhaps, months to cure the disease."

Dr. D. advises to fill the nerve cavity before the abscess is healed. An abscess may be, and often is successfully treated after the canal is closed; yet the cure is much more readily effected while it is still left open. By filling it, we shut ourselves out from the most convenient mode of access to the disease. Judicious treatment through the nerve cavity will arrest the disease, in a large majority of cases, without resort to the heroic treatment described in the sentence beginning with, "Plunge in the lancet, etc."

Dr. M. gives a case in which there seemed to be sensibility remaining in the canal after the nerve had been entirely removed. This is easily explained. The dental nerve in such cases is obliterated but little or no farther than the extremity of the fang. It is liable to irritation from diseased action in the vicinity of its present termination; and the sensation resulting from this irritation will always be felt at its original termination. The brain takes no cognizance of the fact that a part of it is lost. Accordingly, "jumping tooth-ache" may be, and often is experienced in a tooth, of which the pulp cavity and canal are filled with gold. It is on this principle that sensation is felt in the fingers or toes, after the amputation of a limb.

About the plate exhibited by Dr. S., we could say more to the purpose, if all the circumstances were stated. The reason the deposit of base metal is only on one side is, that it was removed from the lingual side by friction, as fast as deposited. Is it mercury? That could be easily known by examination. Had the patient the natural teeth in the lower jaw? And were any of them filled with a base metal? Though not very old in the profession, we have seen quite a number of similar cases, and in each, the deposit was due to the galvanic action resulting from the presence of the two metals, excited by the fluids of the mouth. Recently our attention was called to a twenty carat plate, covered at one side of the mouth with a base metal. We at once suggested the presence of amalgam fillings. The dentist in whose hands it occurred stated that there were none in the mouth. We good-naturedly insisted that there were; and, on a more careful examination, he found two in the lower molars of the affected side. That a second plate was not similarly affected, may be owing to the fact that the first exhausted the supply of base metal, or that its surface had become so oxydized that galvanic currents were no longer excited.

Dr. G. tells us of a patient who was not conscious of having taken mercury, "but it was quite evident she had." We have no faith in the theory that the system of a patient, who had taken so little mercury as to be unconscious of it, would become so impregnated with that drug, as to contaminate a plate inserted in the mouth. The inference is too much like that of the Western "steam doctor," who had salivated a patient with a nostrum, of whose composition he was ignorant. "Madam," says he, "that's the effect of the merc'ry that's been in your system." "It can't be, doctor; I never took any." "Yes, but your parents did; and its effects don't stop at one generation." "My parents did not; my father and grandfather were Indian doctors." After scratching his pate for a new idea, the doctor resumed, "Look here, don't you sometimes eat beef?" "Yes." "Ah, that explains it. The farmers will go to these pizen doctors for medicine to kill the lice on their calves; and they always give them 'precipity' or sublimate, and it gets into their system, and you've eaten some of that kind of beef."

The report of Dr. D. on Dr. Weiber's gold, is rather the richest thing in the whole proceedings. "The gold was about the same as manufactured in this country some six years ago, and *rejected* as not adapted to the wants of the profession." And therefore, "They recommend the Convention to confer an honorary degree upon Dr. Weiber." What for? *For*

*being six years behind the times?* Is it *honorable* for Europeans to be six years behind Americans? "They recommend the *Convention* to confer an honorary degree." A mass meeting, without a charter, without even *a constitution*, is recommended to confer an honorary degree, in acknowledgment of a specimen of gold, of no use and six years behind the times; and it agrees to do it. "The report was accepted and adopted." In plain Anglo-Saxon, all this is very funny.

In conclusion, we will only add that no member of the profession can carefully read the report of discussions without deriving profit from it. If the programme of discussion had been published previous to the meeting, doubtless there would have been less wandering from the questions on hand. But, after all, these wanderings may have been profitable. These Conventions give stronger evidence of the vitality and progressive spirit of the profession than anything else; and we look forward with bright anticipations to its first meeting in the West. W.

